# Author reply to “reporting standards for cerebrospinal fluid studies: more transparency of laboratory data is needed” by JL Frater

**DOI:** 10.1007/s15010-023-02134-4

**Published:** 2023-11-10

**Authors:** Susanne Dyckhoff-Shen, Mathias Bruegel, Uwe Koedel, Matthias Klein

**Affiliations:** 1 Department of Neurology, Ludwigs Maximilians University (LMU) Hospital, Munich, Germany; 2 Institute of Laboratory Medicine, Ludwigs Maximilians University (LMU) Hospital, Munich, Germany; 3 Emergency Department, Ludwigs Maximilians University (LMU) Hospital, Munich, Germany

We thank JL Frater for his letter with important critical remarks on possible preanalytical issues and the specific laboratory techniques used that might influence the results of cerebrospinal fluid (CSF) white cell counts [1].

The preanalytical time is indeed critical for the evaluation of CSF parameters as cells might undergo autolysis with prolonged time periods and, in consequence, the determined cell counts would be falsely low: In a time-dependent analysis, the results of cell counts decreased significantly as early as 60 min after CSF was obtained [2]. Granulocytes and macrophages seem to be the first cell types to decrease in numbers; the amount of large lymphocytes diminishes 90 min after CSF is obtained. At LMU Hospital, Munich, where our study was performed, the central laboratory that performs the cerebrospinal fluid analysis is located in the same building as the emergency department and all clinical wards. A pneumatic tube transportation system connects all clinical sites with the laboratory which ensures quick transport of blood and CSF samples to the laboratory. In this context, it is important to note that recent studies suggest that pneumatic tube transportation systems can be used without clinically significant impacts on the results of cell counts in the CSF (although a minor effect of such transportation systems on certain CSF parameters cannot be completely ruled out) [3, 4]. Our central laboratory is operated 24/7 and CSF analysis is offered immediately at any time. Therefore, time intervals between acquiring and analyzing CSF samples are usually low. Despite this optimal setup, a significant number of cells was found to be destroyed in n = 4 CSF samples of our study [5]. We are unable to determine the actual reason for this, but we assume that CSF was sent to the laboratory with delay in these cases. However, as the portion of such incidences was low (0,3% of all cases), we consider it unlikely that preanalytical time periods significantly affected the results of our study [5].

Indeed, the laboratory techniques used for the assessment of the CSF cell count are crucial, especially in low CSF cell counts below 20 cells / µl [1]. In such cases, the gold standard remains manual cell counting, e.g. using the Fuchs-Rosenthal chamber. In consequence, at the central laboratory of the LMU Hospital, Munich, all samples that show a mild pleocytosis (< 20 cells / µl) are routinely re-assessed by manual cell counting using the Fuchs-Rosenthal chamber, and only CSF samples with automated cell counts above 20 cells / µl are not routinely counted manually. Furthermore, the differential of CSF samples is exclusively and routinely performed only manually by microscope; automated cell analyzers are not in use for this application.

In summary, we would like to support the remarks raised by JL Frater and strongly encourage researchers and physicians to keep preanalytical and analytical issues in mind when the results of CSF analysis are interpreted. Concerning the data used in our study, a relevant impact of such effects on our findings seems unlikely.

## Data Availability

The data that support the findings of this study are available from the corresponding author upon reasonable request.
